# Co-Regulation Mechanism of Host p53 and Fos in Transcriptional Activation of ILTV Immediate-Early Gene *ICP4*

**DOI:** 10.3390/microorganisms12102069

**Published:** 2024-10-16

**Authors:** Zheyi Liu, Xuefeng Li, Lu Cui, Shufeng Feng, Zongxi Han, Yu Zhang, Shengwang Liu, Hai Li

**Affiliations:** 1 Division of Avian Infectious Diseases, State Key Laboratory of Veterinary Biotechnology, National Poultry Laboratory Animal Resource Center, Harbin Veterinary Research Institute, The Chinese Academy of Agricultural Sciences, Harbin 150069, China; liuzy0701@126.com (Z.L.); lixuefeng57@126.com (X.L.); cuilu099@163.com (L.C.); hanzongxi@caas.cn (Z.H.); 2 School of Basic Medical Sciences, Translational Medicine Institute, Key Laboratory of Environment and Genes Related to Diseases of the Education Ministry, Xi’an Key Laboratory of Immune Related Diseases, Xi’an Jiaotong University Health Science Center, Xi’an Jiaotong University, Xi’an 710061, China; feng_xjtu0204@163.com (S.F.); zhangyu2022@xjtu.edu.cn (Y.Z.)

**Keywords:** ILTV, transcriptional regulation, p53, *ICP4*

## Abstract

Infectious laryngotracheitis virus (ILTV) exhibits a cascade expression pattern of encoded genes, and *ICP4* is the only immediate-early gene of ILTV, which plays a crucial role in initiating the subsequent viral genes. Therefore, studying the transcriptional regulation mechanism of *ICP4* holds promise for effectively blocking ILTV infection and spread. Host transcriptional factors p53 and Fos are proven to regulate a variety of viral infections, and our previous studies have demonstrated their synergistic effects in regulating ILTV infection. In this study, we constructed eukaryotic expression vectors for p53 and Fos as well as their specific siRNAs and transfected them into a chicken hepatoma cell line. The results showed that knocking down p53 or Fos significantly inhibited *ICP4* transcription, while overexpressing p53 or Fos had an opposite effect. A further CoIP and ChIP-qPCR assay suggested p53 and Fos physically interacted with each other, and jointly bound to the upstream transcriptional regulatory region of *ICP4*. To elucidate the specific mechanisms of p53 and Fos in regulating *ICP4* transcription, we designed p53 and Fos protein mutants by mutating their DNA binding domains, which significantly reduced their binding ability to DNA without affecting their interaction. The results showed that Fos directly bound to the promoter region of *ICP4* as a binding target of p53, and the p53–Fos protein complex acted as a transcriptional co-regulator of I*CP4*. Studying the transcriptional process and regulatory pattern of *ICP4* is of great significance for understanding the molecular mechanism of ILTV infection, and thus for finding effective methods to control and prevent it.

## 1. Introduction

Herpesviruses are a large family of viruses that infect both humans and animals. The genome isolated from herpesviruses consists of linear double-stranded DNA, transcribed by host RNA polymerase II [1]. During herpesvirus infection, viral gene expression is temporally regulated; namely, the earliest expressed genes regulate the later expressed ones [2]. Based on their temporal expression and dependence on certain gene products, viral genes are classified into three categories, including immediate-early genes, early genes, and late genes [3]. In general, the products of immediate-early genes are mostly transcription factors; early genes encode enzymes involved in nucleotide metabolism and DNA replication, as well as some envelope glycoproteins; late genes partially or entirely rely on viral DNA replication, typically encoding structural proteins involved in virus particle assembly and other proteins [3]. Therefore, deciphering the transcriptional regulation of immediate-early genes of herpesviruses is critical for understanding and preventing herpesvirus infection.

Analysis of the promoter regions of *RTA*, *ORF45*, and *K8* genes of human herpesvirus type 8 (HHV8/KSHV) revealed that activating these viral immediate-early genes, in some situations, relies on host transcription factors, such as c-Fos, c-Jun, Sp1, CREB, C/EBP, c-Myc, and ATF-2 [4,5,6,7,8,9]. During KSHV lytic replication, the expression level of ORF45 increases when the host Fos binds to the promoter region of the viral gene [10]. As is known, Fos is one of the members of the activator protein 1 (AP-1) family, and since its first identification as a DNA-binding protein, Fos has been recognized as an important regulator involved in various biological processes, including herpesvirus invasion and pathogenesis [11]. Interestingly, our previous studies on *Iltovirus gallidalpha1*, also known as *Infectious laryngotracheitis virus* (ILTV), a member of the *Orthoherpesviridae* family and *Alphaherpesvirinae* subfamily, showed that knocking down the host *Fos* gene significantly reduced ILTV replication and spread, while Fos overexpression worked conversely [12,13]. Moreover, our studies suggested that host p53 also played a pivotal role in regulating ILTV replication [13]. p53, a crucial tumor suppressor, was initially discovered as a binding partner of the SV40 large T-antigen [14], while some recent studies also identified it as a host cell response protein, which regulates cellular signaling pathways involved in innate immune control, cell cycle, DNA repair, apoptosis, etc., to cope with a wide range of stress factors including viral infection [15,16]. It is now known that host p53 participates in multiple virus infection processes; however, its specific biological functions depend on the type of virus and even the stage of infection [17,18,19]. As for some viruses, a decreasing host p53 level promotes their replication ability, such as *Hepatitis C virus* (HCV), *Influenza A virus* (IAV), *Japanese encephalitis virus* (JEV), and *Vesicular stomatitis virus* (VSV) [15,19,20,21]. But some other viruses have evolved certain mechanisms to resist the negative effects of p53, and even p53 has become necessary for their replication. For example, knocking down host p53 impairs the replication ability of *Herpes simplex virus type 1* (HSV-1) and its associated central nervous system symptoms [22,23]. It has also been reported that knocking down p53 inhibits the replication of *Human cytomegalovirus* (HCMV) and porcine circovirus type 2 in host cells [24,25].

Avian infectious laryngotracheitis virus (ILTV) belongs to the subfamily of *Alphaherpesvirinae*. Upon ILTV infection, the virus quickly establishes latent infection in the host’s trigeminal nerve node, and there is currently no effective method to clear it out [26]. When the immunity of infected chicken decreases, the virus periodically reactivates from the latent period and replicates, which is the reason for the repeated outbreaks of infectious laryngotracheitis in ILTV-infected chicken flocks. Like other herpesviruses, the expression pattern of ILTV-encoded genes is temporally regulated. *ICP4* is the only immediate-early gene of ILTV, and its encoded product plays an important regulatory role in the transcription of subsequent viral early and late genes. Our previous study has shown that both host p53 and Fos play important roles in regulating *ICP4* transcription [13]. However, the relationship between the two factors and how they regulate *ICP4* transcription remain unclear. Therefore, this study aims to elucidate the molecular mechanism by which host p53 and Fos regulate the transcription of the immediate-early gene *ICP4* upon ILTV infection.

## 2. Materials and Methods

### 2.1. Plasmids, Cell and Virus Strain

Both pCAG-HA and pCAG-Flag expression plasmids were generously provided by the Harbin Veterinary Research Institute of CAAS (Harbin, China). The firefly luciferase reporter plasmid (pGL3-basic vector) and control Renilla luciferase plasmid (pRL-TK) were purchased from the Beyotime Biotechnology Company (Beyotime, Shanghai, China).

Chemically immortalized leghorn male hepatoma (LMH) cells were cultured in Dulbecco’s Modified Eagle’s Medium (DMEM, Sigma, Burlington, MA, USA), supplemented with 10% fetal bovine serum (FBS, Sigma, Burlington, MA, USA), 100 U/mL penicillin and streptomycin and 2 mM L-glutamine (Sigma, Burlington, MA, USA). The cells were maintained in a 5% CO_2_ atmosphere at 37 °C.

The ILTV-LJS09 strain (GenBank Accession No. JX458822) was graciously provided by the Harbin Veterinary Research Institute of CAAS (Harbin, China). This virulent strain has the ability to propagate efficiently in LMH cells, rendering them a suitable host for an ILTV infection study [27].

### 2.2. RNA Extraction and RT-qPCR

Total RNA was extracted from LMH cells using RNAiso Plus (Takara Biotechnology, Dalian, China) according to the manufacturer’s procedure. A NanoDrop microvolume spectrophotometer (Implen GmbH, Munich, Germany) was used to assess the concentration and quality of RNA. Oligo 7 (version 7.6.0, Molecular Biology Insights, Inc., Colorado Springs, CO, USA) was used for primer design, and the information on primers is presented in Table 1. A One-Step TB Green PrimeScript RT-PCR Kit II was used for RT-qPCR and absolute quantitative PCR (Takara Biotechnology, Dalian, China) according to the manufacturer’s procedure. Data were calculated with 2^−ΔΔCT^ method and results were presented as a Log2 fold change or fold change. The standers of absolute quantitative PCR were prepared by cloning the PCR products of gC genes of ILTV into the pMD18-T plasmid (Takara Biotechnology, Dalian, China) according to the manufacturer’s instruction.

### 2.3. RNA Interference

Short interfering RNAs (siRNAs) that specifically recognize sequences in Fos mRNA (siFos: 5′-GGAUCCGCCGGGAGAGGAA-3′), p53 mRNA (sip53: 5′-GCUGCUUCGAGGUGCGCGU-3′) and a negative control siRNA (siControl: 5′-UUCUCCGAACGUGUCACGUTT-3′) were used for RNA interference according to the previous description [11,13]. Subconfluent LMH cells seeded in 24-well plates were transfected with 5 pmol siRNA using Lipofectamine 8000 (Beyotime, Shanghai, China) according to the manufacturer’s instruction. The siRNA-transfected cells were incubated for 24 h and then used for interference efficiency assay or ILTV infection.

### 2.4. Plasmid Construction and Transfection

The coding sequences of chicken *p53* and *Fos* were amplified from the LMH cell cDNA, and the primer information is included in Table 2. The PCR product was purified and digested with Xho I and BamH I (NEB, Ipswich, MA, USA). To generate pCAG-Fos-HA and pCAG-p53-Flag plasmids, the purified PCR product was cloned into the pCAG-HA or pCAG-Flag vector with an In-Fusion^®^ Snap Assembly Master Mix (TaKaRa, Dalian, China).

We engineered p53 and Fos proteins with specific mutations in their DNA binding domains based on previous studies [28,29,30]. Two fully complementary primer pairs were designed for introducing site-directed mutations at the 160th amino acid of p53 (p53-R160H) and the 143rd amino acid of Fos (Fos-R143V) (Table 2). Using p53-F or Fos-F as the forward primer, pm1-R or Fm1-R as the reverse primer, and p53 or the Fos eukaryotic expression vector as the template, the first segment sequence was amplified via PCR. Similarly, using pm1-F or Fm1-F as the forward primer and pm1-R or Fm1-R as the reverse primer, the second segment sequence was amplified. Then, the two sequences were fused, purified, and ligated to a linearized vector, resulting in the pCAG-pm1-Flag or pCAG-Fm1-HA expression vector.

DNA sequence information on *ICP4* (No. JX458822) and *Fos* (No.396512) was retrieved from the GenBank database. The binding sites validated by ChIP-qPCR within 2000 bp upstream of the transcription start sites of these two genes were cloned for pGL3-ICP4-Luc and pGL3-Fos-Luc plasmid construction (Table 2). The pGL3-basic vector underwent digestion with *Xho I* and *Kpn I* enzymes for linearization, and the linearized pGL3-basic vector and cloned promoter sequences were linked using an In-Fusion cloning system (Takara, Dalian, China) according to the manufacturer’s instruction. All PCR products were amplified with KOD-Plus-Neo (TOYOBO, Osaka, Japan) and all DNA constructs used in this study were verified by sequencing.

For plasmid transfection, we added 1 µg plasmid to each well of the 24-well plate and used PEI transfection reagent following the manufacturer’s instruction (R0531, Thermo Scientific, Rockford, IL, USA).

### 2.5. Western Blot Analysis

Western blotting was performed according to our previous description [31]. Briefly, cells were washed with ice-cold PBS and soluble proteins were extracted with RIPA buffer (Beyotime Biotech, Shanghai, China) according to the manufacturer’s protocol. The protein concentration of each sample was determined using a BCA Kit (Beyotime Biotech, Shanghai, China), and an equal amount of protein was separated by SDS-PAGE, followed by a transmembrane onto a nitrocellulose membrane (Millipore, Burlington, MA, USA). The membrane was blocked with 5% non-fat milk for 2 h at room temperature and incubated with primary antibodies overnight at 4 °C. Then the membrane underwent three washes with TBST, followed by 1 h incubation with the corresponding secondary antibody. HA and Flag antibodies were purchased from the Beyotime Biocompany (Beyotime Biotech, AF2858, AF2852, Shanghai, China) and tubulin antibody was purchased from the Sigma biocompany (Sigma-Aldrich, T6074, St. Louis, MO, USA).

### 2.6. Co-Immunoprecipitation (CoIP) Assay

Eukaryotic expression vectors of p53 and Fos were co-transfected into LMH cells. Cells with or without an ILTV infection were lysed on ice with IP-grade RIPA lysis buffer (Beyotime Biotech, Shanghai, China) for 30 min at indicated time points. Protein quantification was conducted using a BCA protein concentration assay kit (Beyotime Biotech, Shanghai, China). One tenth of the protein samples were taken as the input and 100 ng of the total protein from each group was selected for an immunoprecipitation experiment. Firstly, protein samples were precleaned with protein A/G agarose beads (Santa Cruz Biotechnology, Santa Cruz, CA, USA) to remove proteins nonspecifically binding to the beads. Then, the precleaned samples were incubated overnight with IP-grade antibody specifically recognizing HA (Beyotime Biotech, AF2858, Shanghai, China) or Flag (Beyotime Biotech, AF2852, Shanghai, China) according to the manufacturer’s instructions. The proteins bound by antibodies were purified with protein A/G agarose beads by centrifugation. The nonspecific binding of antibodies to proteins was determined with an isotype control antibody. Finally, the samples were analyzed using Western blot.

### 2.7. Chromatin Immunoprecipitation (ChIP) Assay

ChIP experiments were conducted according to a previous publication with some modifications [32]. LMH cells were fixed in 1% formaldehyde for 10 min and then with 0.125 M glycine. Each ChIP experiment was performed with sheared chromatin samples from LMH cells (5 × 10^6^ cells) using 5 µg of antibodies specifically recognizing HA or Flag. IgG1 was used as the isotype control antibody. Protein A/G PLUS-agarose beads were used for the pull-down according to the manufacturer’s instructions. The immunoprecipitated DNA was purified using a QIAquick PCR Purification Kit (QIAGEN, Valencia, CA, USA). The gene promoter region was detected by qRT-PCR using specific primers, and the primers used for the ChIP-qPCR assay are shown in Table 3 and our previous publication [33].

### 2.8. Luciferase Assay

LMH cells at 80% confluence were transfected in 48-well plates with 100 ng of pGL3-Basic vector, 50 ng of pRL-TK and 100 ng of effector plasmid vector or effector plasmid expressing a *Fos* or *p53* gene using PEI (Invitrogen, Carlsbad, CA, USA). The cells were lysed and subjected to luciferase assays at 24 h post transfection using the Dual-Luciferase Reporter Assay System (Promega, Madison, WI, USA) according to the manufacturer’s instructionss. The luciferase activity was determined with a Multiscan Spectrum (Enspire, PerkinElmer, Waltham, MA, USA). The relative luciferase activity was calculated by dividing the firefly luciferase activity by the Renilla luciferase activity.

### 2.9. Bioinformatic and Statistical Analysis

Gene promoter sequence information was obtained from the GenBank database. The 2 kb region upstream of the transcriptional start site was used for searching transcription factor binding sites. The motif position frequency matrix (PFM) of binding sites was retrieved from the Jaspar database version 2024 (http://jaspar.genereg.net/, accessed on 22 January 2024) [33].

Protein structure modeling was performed using the online sites I-TASSER server (https://zhanggroup.org/I-TASSER/, accessed on 13 September 2023) [34,35,36]. Protein interaction likelihood and site prediction was performed using the online website Vakser Lab (https://vakserlab.ku.edu/, accessed on 7 October 2023) [37]. All protein structures were visualized using pymol software (PyMOLTM Molecular Graphics System, Version 2.6.0a0). Protein interaction sites were visualized by PDBePISA (https://www.ebi.ac.uk/pdbe/pisa/, accessed on 7 October 2023) [38,39].

Statistical analysis was performed using a GraphPad software suite (GraphPadPrism for Windows version 8.0, SPSS, San Diego, CA, USA, www.graphpad.com, accessed on 4 March 2022). Data from several experiments are presented as mean ± standard deviation (SD). The significance of differences between two groups was determined with a two-tailed Student’s *t* test. One-way or two-way analysis of variances with a Bonferroni correction was employed for multi-group comparison. * *p* < 0.05 and ** *p* < 0.01 indicate the levels of significance.

## 3. Results

### 3.1. Both p53 and Fos Upregulate the Transcription Level of ICP4

**Figure 1:**
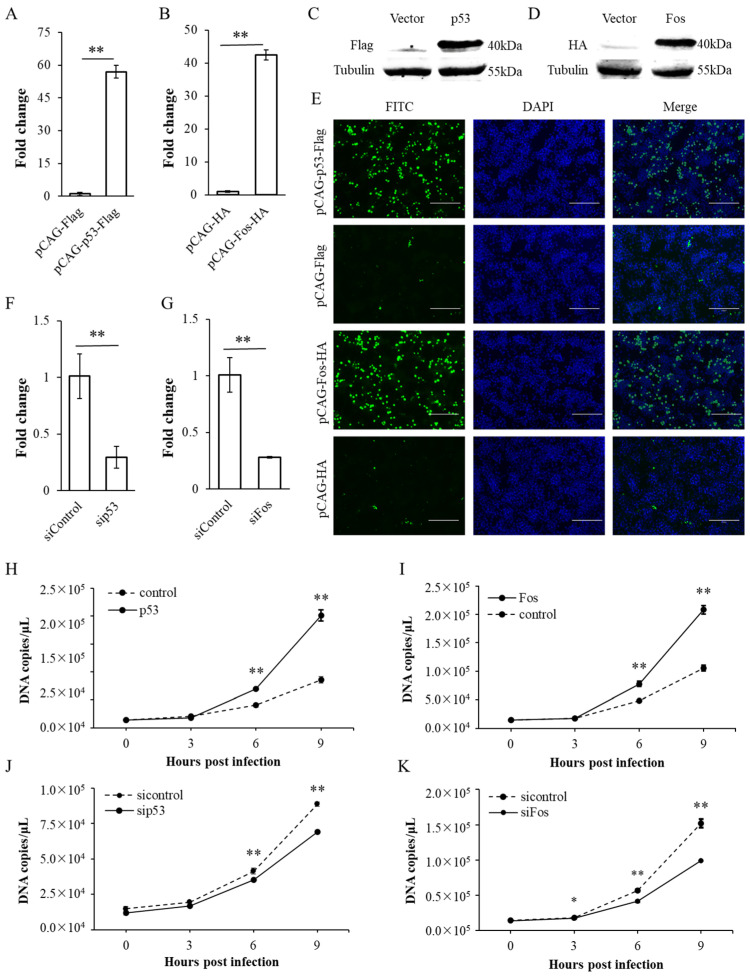
Effects of p53 and Fos overexpression and knockdown on the transcription of *ICP4.* (**A**–**E**) 24 h after instantaneous transfection of pCAG-p53-Flag and pCAG-Fos-HA into LMH cells, the overexpression efficiency of p53 and Fos was analyzed on mRNA level by RT-qPCR (**A**,**B**) and on protein level by Western blot (**C**,**D**) and immunofluorescence (**E**). The scale bar indicates 150 µm. (**F**,**G**) The knockdown efficiency of p53 and Fos was analyzed on mRNA level by RT-qPCR 24 h after sip53 and siFos transfection into LMH cells. (**H**,**I**) 24 h after transfection with pCAG-Flag, pCAG-p53-Flag or pCAG-HA, pCAG-Fos-HA, LMH cells were infected with ILTV (MOI = 1). The transcription level of ILTV immediate-early gene *ICP4* was detected at the indicated time points by absolute quantitative PCR with standard curve method. (**J**,**K**) 24 h after transfection with sicontrol, sip53 or siFos, LMH cells were infected with ILTV (MOI = 1). The transcription level of ILTV immediate-early gene *ICP4* was quantitatively detected at the indicated time points. Data in (**A**,**B**,**F**–**K**) are presented as the mean ± SD, *n* = 3. * *p* < 0.05 and ** *p* < 0.01 indicate the levels of significance.

The constructed pCAG-p53-Flag and pCAG-Fos-HA eukaryotic expression vectors were transfected into the LMH cell line, and 24 h later, the overexpression efficiency was detected by RT-qPCR, Western blot and indirect immunofluorescence. The experimental results showed that mRNA levels of p53 and Fos in the transfected cells were significantly increased (Figure 1A,B), along with specific bands and significant green fluorescence observed in the cells (Figure 1C–E). Therefore, pCAG-p53-Flag and pCAG-Fos-HA were successfully expressed in the transfected LMH cells. Then, the LMH cell line was transfected with small interfering RNA (siRNA) targeting chicken p53 (sip53) and Fos (siFos), and 24 h later, the interference efficiency was detected by RT-qPCR. The experimental results showed that compared with the sicontrol group, the transcription levels of p53 and Fos in the sip53 and siFos interference groups were significantly decreased (Figure 1F,G).

By overexpressing or interfering host p53 and Fos, we studied the impact of these genes on the transcription level of the ILTV immediate-early gene *ICP4*. Some 24 h after transfection of p53 and Fos expression vectors or small interfering RNA, the cells were infected with ILTV, and then the transcription level of *ICP4* was detected at 0 h post infection (hpi), 3 hpi, 6 hpi, and 9 hpi. According to the results of absolute quantitative PCR, the transcription level of *ICP4* increased gently from 0 hpi to 3 hpi and significantly from 3 hpi to 9 hpi. Compared with the control group, the overexpression of p53 and Fos significantly increased the transcription level of *ICP4* at 6 hpi and 9 hpi, while knocking down host p53 or Fos significantly inhibited the transcription level of *ICP4* (Figure 1H–K). These results suggest that both p53 and Fos positively regulate the transcription level of *ICP4*.

### 3.2. p53 Promotes Fos Transcription Independent of Binding to Its Promoter Region

**Figure 2:**
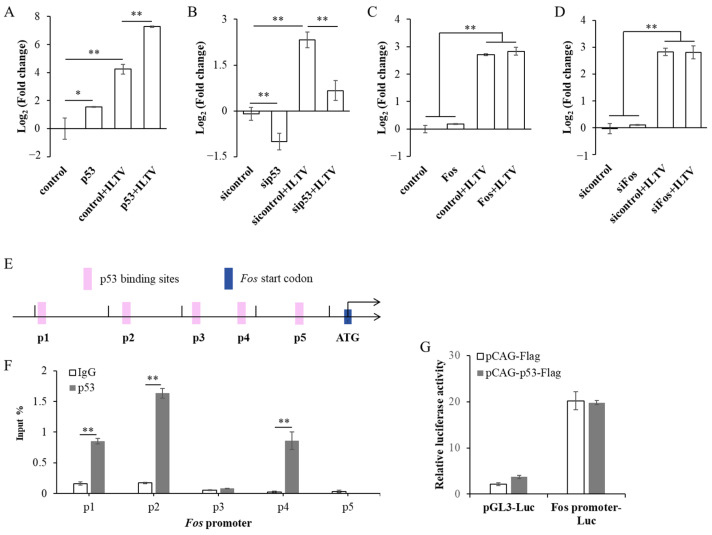
p53 promotes *Fos* transcription and directly binds to the promoter region of *Fos*. (**A**,**B**) Effect of overexpression or knockdown of p53 on *Fos* transcription was assayed by RT-qPCR. (**C**,**D**) Effect of overexpression or knockdown of Fos on *p53* transcription was assayed by RT-qPCR. (**E**) Prediction of the putative p53 DNA binding sites within the 2000 bp upstream region of *Fos* gene using our previous ChIP-sequencing data. (**F**) The binding level of p53 on the predicted sites was validated by ChIP-qPCR. (**G**) The transcriptional activity of p53 was assayed by dual-luciferase reporter assay. Data in (**A**–**D**,**F**,**G**) are represented as mean ± standard deviation, *n* = 3. * *p* < 0.05 and ** *p* < 0.01 indicate the levels of significance.

Considering the consistence of p53 and Fos in regulating the transcription of *ICP4*, we next investigated the mutual regulatory relationship between the two genes. Firstly, LMH cells were transfected with sip53 or pCAG-p53-Flag and infected with ILTV 24 h later. At 9 hpi, RT-qPCR was used to detect the transcription level of *Fos*. As shown in Figure 2A,B, ILTV infection significantly increased the transcription level of *Fos*. Regardless of ILTV infection or non-infection, p53 overexpression promoted the transcription of *Fos* (Figure 2A), while p53 knockdown had the opposite effect (Figure 2B). Next, in order to clarify the effect of Fos on the transcription level of *p53*, LMH cells were transfected with siFos or pCAG-Fos-HA and infected with ILTV 24 h later. At 9 hpi, RT-qPCR was used to detect the transcription level of *p53*. As shown in Figure 2C-D, ILTV infection significantly increased the transcription level of *p53*, but Fos overexpression or knockdown had no significant effect on the transcription level of *p53* regardless of ILTV infection or non-infection. These results indicate that although ILTV infection activates the transcription of host Fos and p53, p53 upregulates *Fos* independent of ILTV infection in chicken LMH cells.

Since p53 can regulate *Fos* transcription, is p53 a direct transcription regulator of *Fos*? To answer this question, we first searched for the conserved motif of p53 within 2000 bp upstream of the transcription initiation site of the *Fos* gene based on our previous ChIP-sequencing data [40], and found five potential target sites (Figure 2E, p1–p5). Therefore, we transfected the pCAG-p53-Flag expression vector into LMH cells and collected cell DNA after 24 h. Sonicated chromatin was used for ChIP, and DNA was purified from ChIP samples for qPCR analysis (ChIP-qPCR). The experimental results showed that p53 bound to sites p1, p2, and p4 in the *Fos* promoter region (Figure 2F). To further elucidate the biological effects of p53 targeting the *Fos* promoter region, we inserted the above-mentioned p53 targets into the eukaryotic expression vector pGL3-Luc containing the firefly luciferase gene sequence (*Fos* promoter-Luc), and performed a dual luciferase reporter gene assay. As shown in Figure 2G, no matter that the cells were transfected with pGL3-Luc vector or *Fos* promoter-Luc vector, p53 overexpression did not significantly affect their luciferase activity, suggesting p53 binding to the above-mentioned *Fos* promoter region was not sufficient to initiate *Fos* transcription. Thus, we inferred that p53 regulates *Fos* transcription maybe with other co-regulatory factors or through indirect pathways.

### 3.3. p53 Indirectly Binds to the Promoter Region of ICP4 as a Transcriptional Regulator

**Figure 3:**
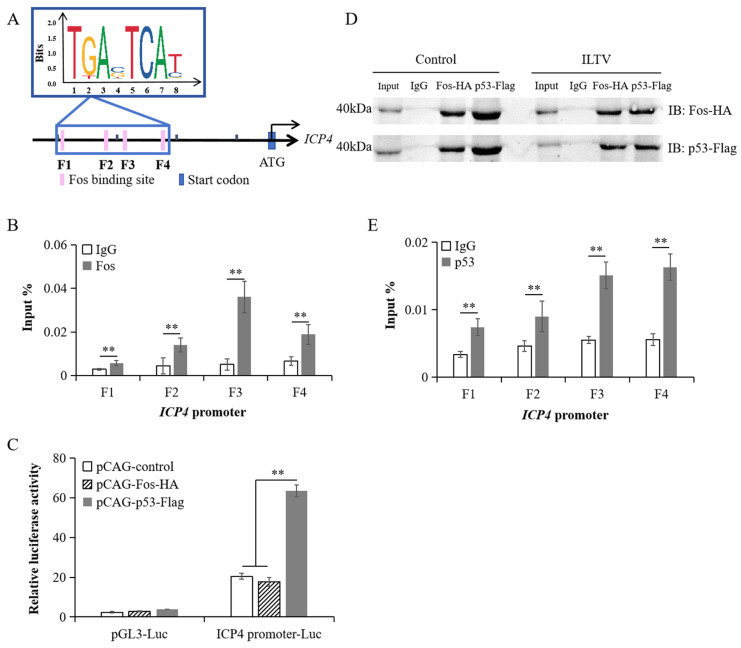
p53 indirectly binds to *ICP4* promoter as a transcriptional regulator. (**A**) Prediction of the putative Fos DNA binding sites within the 2000 bp upstream region of *ICP4* gene using Jasper database. (**B**) The binding level of Fos on the predicted sites was validated by ChIP-qPCR. (**C**) The transcriptional activity of Fos was assayed by dual-luciferase reporter assay. (**D**) Co-IP of p53 and Fos in LMH cells with or without ILTV infection (MOI = 1) using antibodies specifically recognizing HA or Flag. IP: Immunoprecipitation; IB: Immunoblotting. (**E**) The binding level of p53 on the predicted sites was validated by ChIP-qPCR. Data in (**B**,**C**,**E**) are represented as mean ± standard deviation, *n* = 3. ** *p* < 0.01 indicates the levels of significance.

Subsequently, we explored the underlying mechanism of Fos regulating *ICP4* transcription. We used the Jasper database to predict the binding sites of Fos within the 2000 bp upstream region of *ICP4*, and found four potential binding sites for Fos in this region (Figure 3A, F1–F4). Then, LMH cells were transfected with the pCAG-Fos-HA expression vector and infected with ILTV (MOI = 1) after 24 h. At 9 hpi, we collected cell DNA. Sonicated chromatin was used for ChIP and DNA was purified from ChIP samples for ChIP-qPCR analysis. The results showed that Fos was significantly enriched at all four sites mentioned above (Figure 3B). However, the results from the dual luciferase reporter gene assay showed that Fos overexpression had no effect on luciferase activity compared to the control group (Figure 3C), indicating that Fos binding to the promoter region of the *ICP4* gene itself is not sufficient to activate *ICP4* transcription. Given p53 could also upregulate the transcription level of *ICP4*, we transfected LMH cells with p53 and Fos eukaryotic expression vectors simultaneously, and the cells infected with ILTV (MOI = 1) after 24 h were set as the ILTV group, while the uninfected cells were set as the control group. At 9 hpi, protein samples were collected from the cells for immunoprecipitation detection and the results showed that regardless of ILTV infection or non-infection, p53 physically combined with Fos in the cells (Figure 3D), indicating p53 and Fos may jointly carry out biological functions. It should be noted that, although the analysis based on the Jasper database did not predict any potential binding sites for p53 within the 2000 bp upstream range of *ICP4*, ChIP-qPCR results showed that p53 could bind to all four binding sites of Fos in the *ICP4* gene promoter region (Figure 3E), indicating that p53 may bind to the promoter region of *ICP4* via Fos. In addition, the dual luciferase reporter gene assay results showed that, unlike the result from Fos overexpression, p53 overexpression significantly promoted luciferase activity (Figure 3C), indicating that p53 is a putative transcriptional regulator of *ICP4*.

**Figure 4:**
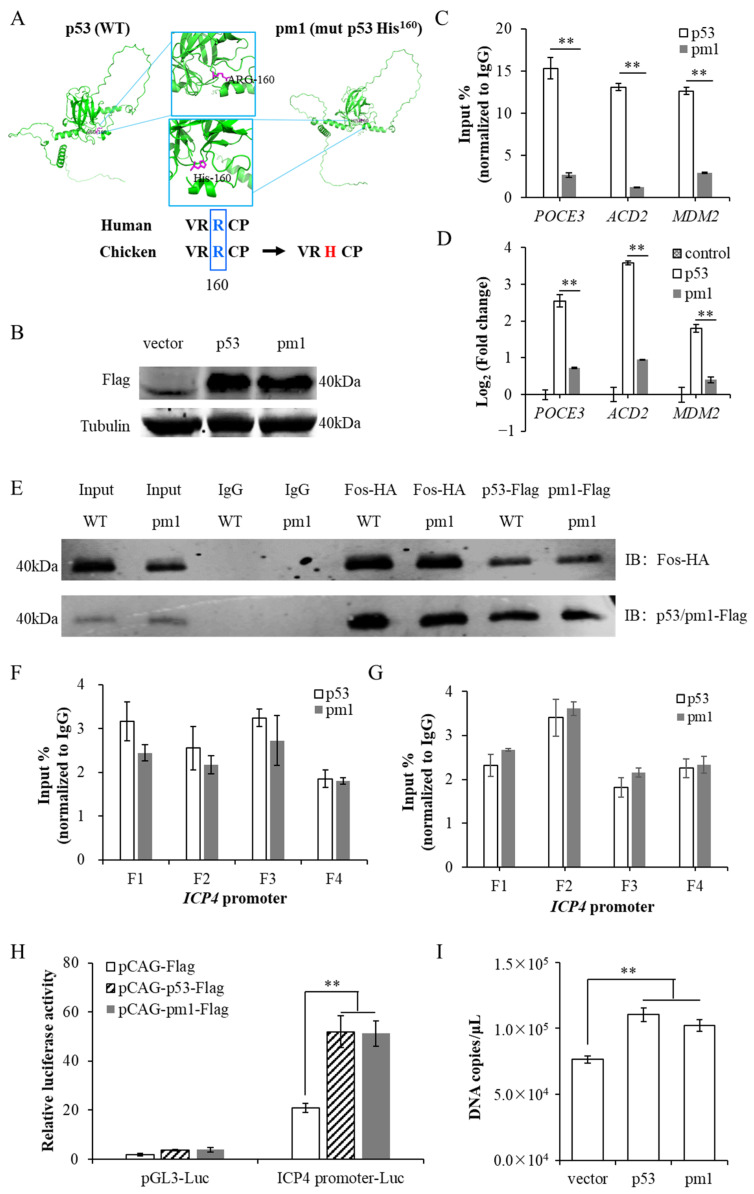
p53 directly binding with DNA is not necessary for transcriptional regulation of *ICP4.* (**A**) Mutation of chicken p53 at the conserved region of DNA binding domain. (**B**) 24 h after instantaneous transfection of pCAG-p53-Flag and pCAG-pm1-Flag into LMH cells, the overexpression efficiency was analyzed by Western blot. Tubulin was used as the inner control. (**C**) The binding of wtp53 and pm1 to the classical p53 target genes was detected by ChIP-qPCR. (**D**) The effects of wtp53 and pm1 on the transcription of classical p53 target genes were assayed by RT-qPCR. (**E**) Co-IP of p53 and Fos in LMH cells with or without p53 mutation using antibodies specifically recognizing HA or Flag. IP: Immunoprecipitation; IB: Immunoblotting. (**F**) The binding of wtp53 and pm1 to *ICP4* promoter was detected by ChIP-qPCR. (**G**) The binding of Fos to *ICP4* promoter upon co-overexpression of wtp53 or pm1 was detected by ChIP-qPCR. (**H**) The transcriptional activities of wtp53 and pm1 were assayed by dual-luciferase reporter assay. (**I**) LMH cells were transfected with pCAG-Flag, pCAG-p53-Flag, or pCAG-pm1-Flag, and 24 h later infected with ILTV (MOI = 1). The transcription level of *ICP4* was quantitatively detected by absolute quantitative PCR with standard curve method. Data in (**C**,**D**,**F**–**I**) are represented as mean ± standard deviation, *n* = 3. ** *p* < 0.01 indicate the levels of significance.

To elucidate the biological function of the p53–Fos protein complex, as well as the molecular mechanism by which p53 regulates *ICP4* transcription, we carried out the following experiments. We first constructed a p53 mutant protein pm1 by mutating the conserved region of the p53 DNA binding domain (Figure 4A). This mutation does not affect p53 protein expression (Figure 4B) and the binding ability with the Fos protein (Figure 4E), but reduces its direct binding ability with DNA and corresponding transcriptional activity (Figure 4C,D). Using pm1, we investigated the necessity of p53 directly binding with DNA for *ICP4* transcriptional regulation. LMH cells were overexpressed p53 and Fos or pm1 and Fos, and were infected with ILTV (MOI = 1) after 24 h. At 9 hpi, we collected cell DNA for ChIP-qPCR detection. The experimental results showed that pm1 enriched at the *ICP4* promoter region with a similar level to wild-type p53 (wtp53), without impacting the enrichment of Fos in the same region (Figure 4F,G). A dual luciferase reporter gene assay further showed that when binding to the *ICP4* promoter region, the transcriptional activity of pm1 was similar to that of wtp53 (Figure 4H). Furthermore, the results from quantitative PCR detection showed that overexpression of either pm1 or wtp53 significantly increased the transcription level of *ICP4* (Figure 4I). From the above data, we can see that p53 protein directly binding with DNA is not necessary for transcriptional regulation of *ICP4*, further confirming that p53 protein regulates *ICP4* transcription by indirectly targeting its promoter region.

### 3.4. Fos Directly Binds to the Promoter Region of ICP4 as a Binding Target of p53

**Figure 5:**
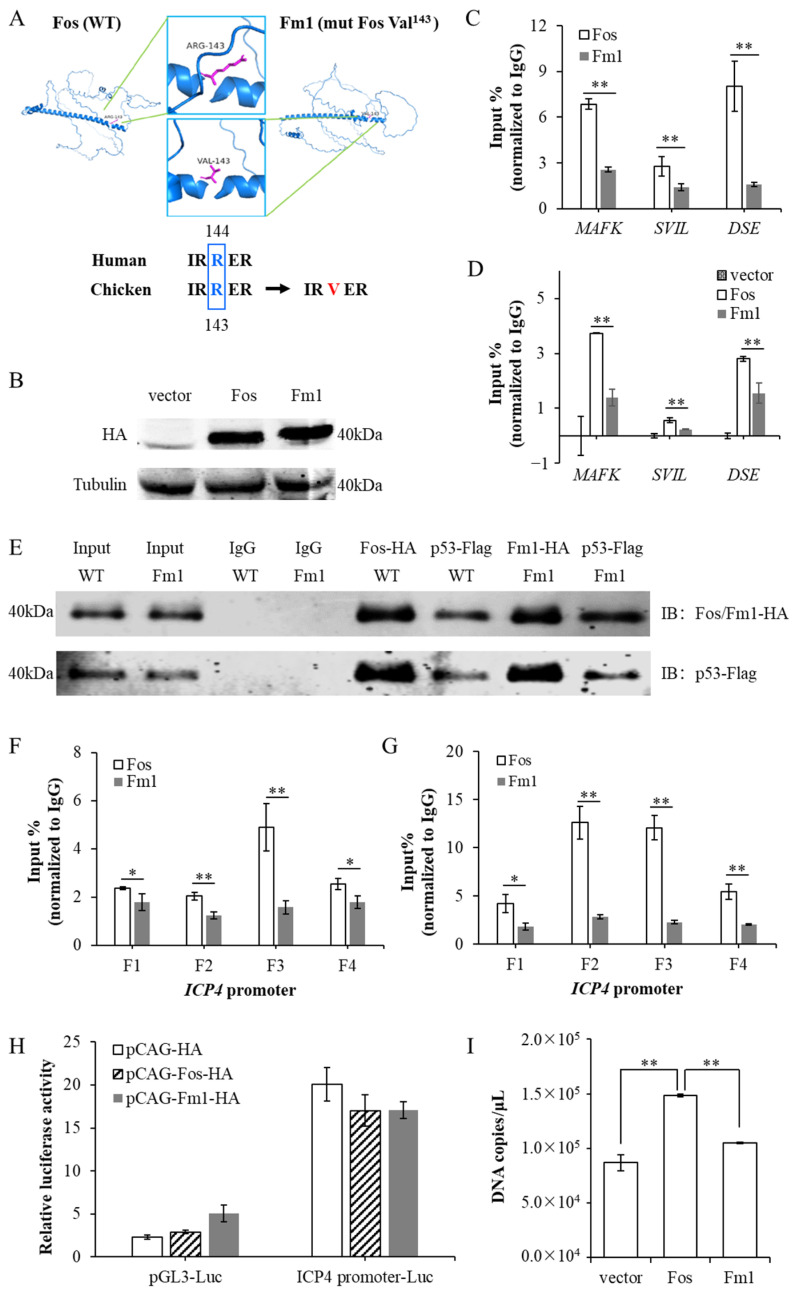
Fos directly binding with DNA is required for the transcriptional regulation of *ICP4* by p53. (**A**) Mutation of chicken Fos at the conserved region of DNA binding domain. (**B**) 24 h after instantaneous transfection of pCAG-Fos-HA and pCAG-Fm1-HA into LMH cells, the overexpression efficiency was analyzed by Western blot. Tubulin was used as the inner control. (**C**) The binding of wide-type Fos and Fm1 to the classical Fos target genes was detected by ChIP-qPCR. (**D**) The effect of wide-type Fos and Fm1 on the transcription of classical Fos target genes was assayed by RT-qPCR. (**E**) Co-IP of p53 and Fos in LMH cells with or without Fos mutation using antibodies specifically recognizing HA or Flag. IP: Immunoprecipitation; IB: Immunoblotting. (**F**) The binding of Fos and Fm1 to *ICP4* promoter was detected by ChIP-qPCR. (**G**) The binding of p53 to *ICP4* promoter upon co-overexpression of wide-type Fos and Fm1 was detected by ChIP-qPCR. (**H**) The transcriptional activities of wide-type Fos and Fm1 were assayed by dual-luciferase reporter assay. (**I**) LMH cells were transfected with pCAG-HA, pCAG-Fos-HA, or pCAG-Fm1-HA, and 24 h later infected with ILTV (MOI = 1). The transcription level of *ICP4* was quantitatively detected by absolute quantitative PCR with standard curve method. Data in (**C**,**D**,**F**–**I**) are represented as mean ± standard deviation, *n* = 3. * *p* < 0.05 and ** *p* < 0.01 indicate the levels of significance.

The above study showed that Fos binds to the promoter region of *ICP4*, while it does not function as an *ICP4* transcriptional regulator (Figure 3B,C); then, what biological effect does this binding have? To answer this question, we carried out the following experiments. We first constructed a Fos mutant protein Fm1 by mutating the conserved region of the Fos DNA binding domain (Figure 5A). This mutation does not affect the Fos protein expression (Figure 5B) and binding ability with p53 protein (Figure 5E), but reduces its direct binding ability with DNA and corresponding transcriptional activity (Figure 5C,D). Using Fm1, we investigated the necessity of Fos directly binding with DNA for *ICP4* transcriptional regulation. LMH cells were overexpressed p53 and Fos or p53 and Fm1, and they were infected with ILTV (MOI = 1) after 24 h. At 9 hpi, we collected cell DNA for ChIP-qPCR detection. Compared with Fos, Fm1 had a much lower binding ability in the promoter region of *ICP4*, and in the meantime, the binding ability of p53 in the same region was impaired (Figure 5F,G). Although dual luciferase reporter gene assay showed that neither Fm1 nor wild-type Fos had transcriptional activity (Figure 5H), a quantitative PCR detection result showed that Fm1 abolished the promoting effect of Fos overexpression on *ICP4* transcription (Figure 5I). In summary, the Fos protein directly binding to the promoter region of *ICP4* is necessary for the transcriptional regulation of *ICP4* by p53, although it itself does not exert a regulating function. That is to say, the p53 protein indirectly targets the *ICP4* promoter region via the Fos protein and thus plays a transcriptional regulatory role.

**Figure 6:**
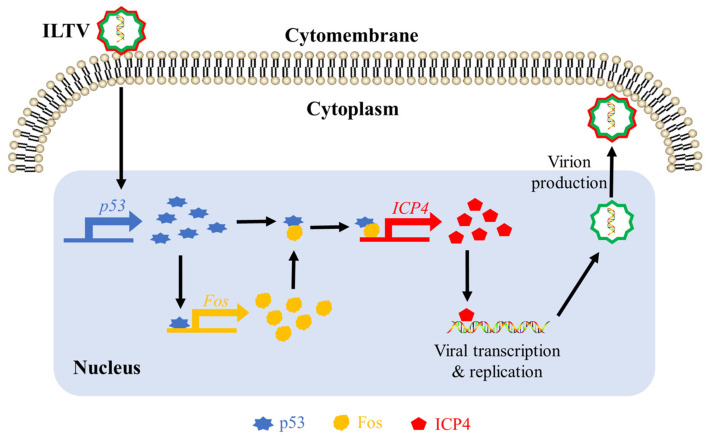
Diagram of host p53 and Fos in transcriptional activation of ILTV immediate-early gene *ICP4*.

In summary, as shown in Figure 6, ILTV infection induces the transcription and expression of host p53, which binds to the promoter region of Fos to activate its expression, and the Fos protein directly binds to the promoter region of ILTV immediate-early gene *ICP4* as a binding target of p53; thus, the p53–Fos protein complex acts as a transcriptional co-regulator of *ICP4*. It is noteworthy that the Fos protein binding to the promoter region of *ICP4* is necessary for the transcriptional regulation of *ICP4* by p53, although it itself does not exert a regulating function.

## 4. Discussion

Unlike human herpesviruses, *ICP4* is the only immediate-early gene expressed during ILTV infection, and its transcription is necessary for initiating the viral gene cascade in the subsequent infectious stages [3]. Studying the transcriptional process and regulatory pattern of *ICP4* is of great significance for understanding the molecular mechanism of ILTV infection, and thus to find effective ILTV control and therapeutic methods.

We previously used metabolomic technology to explore the metabolic patterns of ILTV during the division stage and identified some key host enzymes involved in nucleotide metabolism and ATP synthesis, which were regulated by p53 and Fos [13,41]. These studies also showed that knocking down the host *p53* or *Fos* gene significantly reduced ILTV replication and spread. Thus, in the recent study, we first explored the impacts of host p53 and Fos on the transcription level of *ICP4*. Then, we studied the binding patterns of p53 and Fos proteins within the *ICP4* promoter region, and by inducing mutations in their conserved sites of DNA binding domains, we further investigated the molecular mechanisms by which p53 and Fos regulated *ICP4* transcription. Our study showed that the binding of Fos alone within the *ICP4* promoter region could not initiate *ICP4* transcription, while it works as a binding target of p53, so that the p53–Fos protein complex acts as a co-regulator of *ICP4* and plays a transcriptional regulatory role.

**Figure 7:**
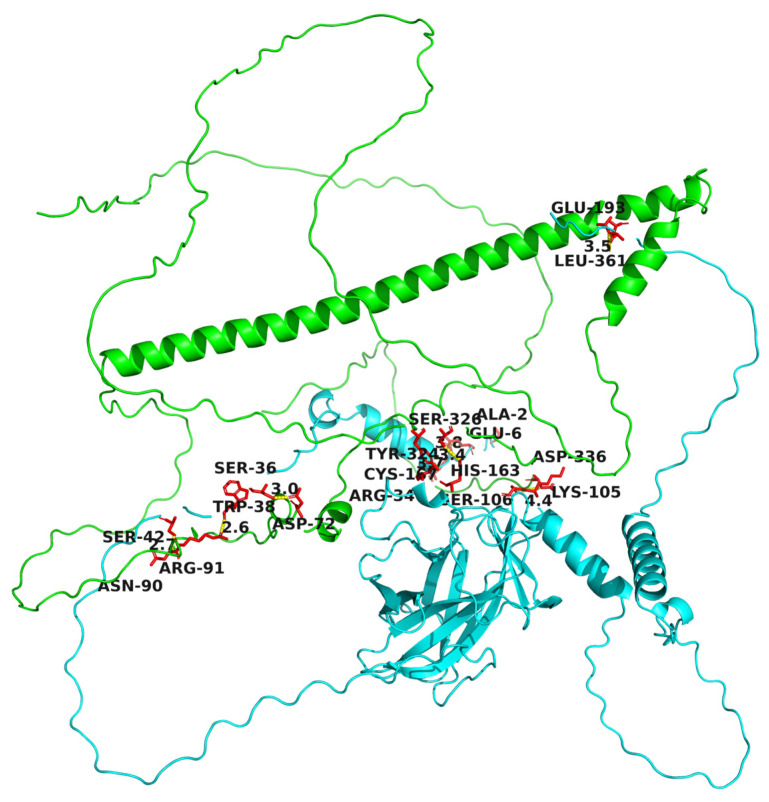
Prediction of the interaction between p53 monomer and Fos monomer.

**Figure 8:**
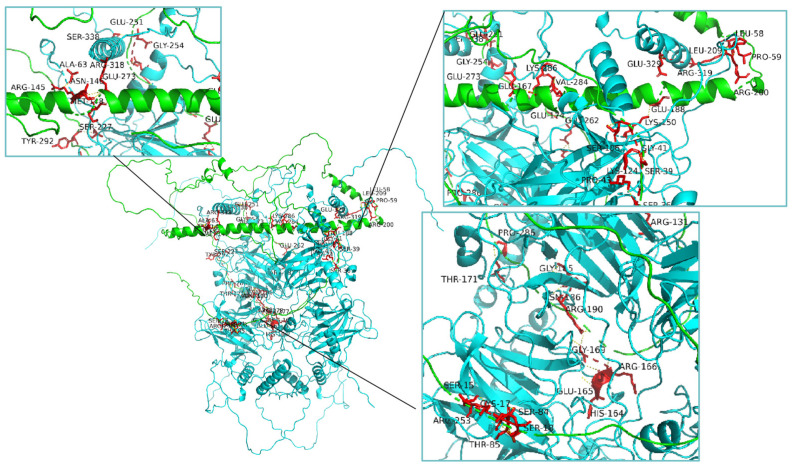
Prediction of interaction between p53 tetramer and Fos monomer.

Since p53 and the Fos protein combine and work as a co-regulator, then how do they interact? To answer this question, we analyzed their protein structures and obtained two different models of interaction. One is the binding between the p53 monomer and the Fos monomer, as shown in Figure 7, and the other is the binding between the p53 tetramer and the Fos monomer, as shown in Figure 8. In fact, the DNA binding region of human p53 is also constituted by the polymerization of four p53 monomers, namely p53 tetramer [42]. When we docked the avian p53 homodimer with each other, we were able to obtain the same tetramer structure. It is worth noting that, during the dimer modeling process of the Fos protein, it was suggested that avian Fos could not form homodimers, which was consistent with previous research on human c-Fos [43]. While whether avian p53 acts as a monomer or a tetramer to perform a DNA binding function, as well as the real binding sites of p53 and Fos, still need further experimental evidence.

Three-dimensional structures of the p53 protein monomer and Fos protein monomer were constructed, respectively, and their binding conformation was predicted. Light green indicates the Fos protein and dark green indicates the p53 protein, and their binding sites are listed and visualized.

Three-dimensional structures of the p53 protein tetramer and Fos protein monomer were constructed, respectively, and their binding conformation was predicted. Light green indicates the Fos protein and dark green indicates the p53 protein, and their binding sites are listed and visualized.

The specific mechanism of transcription initiation of ILTV immediate-early gene *ICP4* is still unclear, while the studies on human herpesvirus HSV-I have shown that VP16 protein, encoded by virus UL48 gene, is involved in the transcriptional regulation of HSV-I immediate-early genes [44]. VP16 has a weak and unstable DNA binding ability; therefore, VP16 primarily forms a complex, by binding to HCF-1 and Oct-1 through the unstructured region within its DNA binding domain, to stabilize its binding to the specific promoter region of target genes. It then recruits transcription factors through its transcription activation domain (TAD) to promote the transcription of target genes [45]. VP16 forms the transcriptional regulatory complex via its conserved DNA binding domain, known as the VP16-induced complex formation domain (VIC), and the complex formed by VP16, HCF-1 and Oct-1 represents VP16-mediated activation of a viral gene cascade expression, which acts as a regulatory switch for two modes of viral infection, namely lytic infection and latent infection. When activated, it promotes the transcription of immediate-early genes, leading to lytic infection, and when inactive, it restricts the transcription of immediate-early genes, allowing HSV-I to maintain a latent infection [46,47,48,49]. Besides forming transcriptional regulatory complexes with HCF-1 and Oct-1, it is unclear whether VP16 has other mechanisms for regulating viral immediate-early gene transcription. Recently, some studies have focused on the spatial interactions between VP16 and its binding proteins, which will help us to understand how the transcriptional activators exert biological functions [44]. In this study, we elucidated the mechanism that hosts p53 and Fos co-regulated viral immediate-early gene *ICP4* during ILTV infection. Given both p53 and Fos are important transcription factors within host cells, investigating whether they are recruited by VP16 is interesting and necessary.

Previous studies have identified *c-Fos* a target gene of p53. Along with p53 activation, the mRNA and protein levels of *c-Fos* significantly increase, and upon DNA damage, p53-dependent induction of *c-Fos* can also be observed in vivo [49]. The first intron of the *c-Fos* gene contains a potential p53 binding site, and p53 dependent transcription activation of *c-Fos* is achieved through binding to different p53 binding elements within this region. It is noteworthy that the ability of p53 to trigger *c-Fos* expression to some extend depends on the cell type [49]. In the LMH cell line, we also observed a significant increase in the *Fos* transcription level after overexpressing p53, and we found a p53 binding site upstream of the avian *Fos* gene. However, in the subsequent dual-luciferase reporter assay, we did not observe significant luciferase activity upon p53 overexpression, which may be due to the lack of certain p53 co-regulatory factors in the LMH cells, which in turn affects the transcriptional regulatory function of p53.

## Figures and Tables

**Table 1:** Information of RT-PCR primers.

Gene Name	Forward Primers (5′-3′)	Reverse Primers (5′-3′)
*β-actin*	GTGGATCAGCAAGCAGGAGT	ATAAAGCCATGCCAATCTCGT
*Fos*	ACAGCCTCACCTACTACCCG	GGTGCAGAAATCCTGCGAGT
*TP53*	ACCAAACGGCACAGCGTCGTC	CACACGCGCACCTCGAAGCAG
*MDM2*	GACAGGAGCAATAGCAGC	AACAGAACCGTGGTCAAA
*DSE*	TTGTGGGACGCTCCTGTA	TTGTGCTGCTCATTGGTA
*MAFK*	CGCTTCCATCTGCTGTTC	CTCCCACTCCCATCCACT
*SVIL*	CTCTGTTTCTTATGGGTAGG	TACAGCCAAGTTCCCTCT
*ACD*	CTCCTGAGAAGACTGGCTACG	GCCTGTTCCAAGGGTTGT
*POLE3*	GACCTCAACCTGCCCAACG	TCGGCTCTTATCTTGTTCCT
*ICP4*	AATGCCATACCCAAACCAACT	AATGACAGCCCACCTAATCCC

**Table 2:** Primers for plasmid construction.

Primer Name	Sequences (5′-3′)	Restriction Recognition Sites
Fos-F	5′-CAAAGAATTCCTCGAGGCCACCATGATGTACCAGGGCTTCGC-3′	Xhol I
Fos-R	5′-CAGCAGATCTGGATCCTATCAAGCGTAGTCTGGGACGTCGTATGGGTACAAGGCCAGCAGGGTGGGGG-3′	BamH I
p53-F	5′-CAAAGAATTCCTCGAGGCCACCATGATG GAATTCCGAACGGCG-3′	Xhol I
p53-R	5′-CAGCAGATCTGGATCCTATCACTTGTCATCGTCGTCCTTGTAATCTTCCTTTTTTTTTCA-3′	BamH I
Fm1-F	5′-GAGGAGGATCCGCGTAGAGAGGAACAAGA-3′	none
Fm1-R	5′-TCTTGTTCCTCTCTACGCGGATCCTCCTC-3′	none
pm1-F	5′-CGAAGTGGTGCGGCACTGCCCCCACCACG-3′	none
pm1-R	5′-CGTGGTGGGGGCAGTGCCGCACCACTTCG-3′	none
ICP4-luc-F	5′-TTTCTCTATCGATAGGTACCACTTCCTTGGCACTCCCAGG-3′	Kpn I
ICP4-luc-R	5′-GATCGCAGATCTCGAGTGGGTAGGTTTCAGCAAAGG-3′	Xhol I
Fos-luc-F	5′-TTTCTCTATCGATAGGTACCCTCTGCTGTGACTTCCCAAG-3′	Kpn I
Fos-luc-R	5′-GATCGCAGATCTCGAGAAGCCCGGAGACACCCACCC-3′	Xhol I

**Table 3:** Primers for ChIP-qPCR assay.

Primer Name	Forward Primers (5′-3′)	Reverse Primers (5′-3′)
Fos-promoter-1	CAACCGGGTGCATGTGATG	CCGCTGAGGCAAGGAAACA
Fos-promoter-2	GCTGCATTTAGCCCGTGAGC	TTTACGGCAACAAACAGAGCC
Fos-promoter-3	GGCGGCAGGGTATAAAGGG	GCTGTTTGCGGGGCCGTGCGGTGCT
Fos-promoter-4	CCGCAGGATTTCTGCACCG	GGGCGACCGAGGAGATGAG
Fos-promoter-5	GCACCTACACCTCCACCTTCG	CCATGCGGTTTGCTACATCTC
DSE-chip	AAGACAGCCATGAGGAAT	GAGGGAAGATGGGTGTAG
SVIL-chip	CACGGTATTATCTAAGTATT	AACCTGCCAGCCCCACAG
MAKF-chip	GCCCCACCACAGCATAGC	ACTGACGGCGTATTCCCT
POLE3-chip	CTGCCCAACGCCGTCATCACC	CCCCGCGCCGAAGATCTCTGA
MDM2-chip	TTTCGGAAGTGCTGTTGTTGCTG	TCTGTAAGGCTGCTTTCCCAT
ACD-chip	CCCACTCACTTCCCAACTCC	TCAGCCTCTGCTTTGTCCTCT

## Data Availability

All the data generated from the current study are included in the manuscript. The raw data are available from the authors without undue reservation.
